# A remote and avatar-based approach for social cognitive impairments in psychotic disorders: A Canadian adaptation of RC2S

**DOI:** 10.1016/j.scog.2026.100462

**Published:** 2026-09-03

**Authors:** Elisabeth Thibaudeau, Elodie Peyroux, Nicolas Franck, Zelda Prost, Martin Lepage

**Affiliations:** a Université Laval, School of psychology, Quebec, Canada; b CERVO Brain Research Centre, Quebec, Canada; c Pôle Hospitalo-Universitaire ADIS, Centre Hospitalier Le Vinatier, Lyon, France; d Centre d'excellence iMIND – Autisme et Troubles du Neurodéveloppement, Centre Hospitalier Le Vinatier, Lyon, France; e Centre Hospitalier le Vinatier, Lyon, France; f Université Claude-Bernard-Lyon-I, Lyon, France; g Dispositif de Réhabilitation Psycho-Sociale, Centre Psychothérapique de l'Ain (CPA 01), Bourg-en-Bresse, France; h Department of Psychiatry, McGill University, Montreal, Canada; i Douglas Research Center, Montreal, QC, Canada

**Keywords:** Social cognitive training, Cognitive remediation, Schizophrenia spectrum disorders, Social cognition, Feasibility, Acceptability

## Abstract

**Background:**

Social cognition is a core impairment in psychotic disorders and is strongly associated with functional outcomes. Although social cognitive training is effective, challenges remain regarding skill generalization and accessibility. This study aimed to develop culturally adapted French- and English-Canadian versions of RC2S and to assess the feasibility and acceptability of remote delivery.

**Methods:**

Fourteen individuals with a psychotic disorder were assessed at baseline, offered 24 twice-weekly RC2S sessions with a therapist over 12 weeks, and reassessed post-intervention. Motivation and therapeutic alliance were assessed through intra-session questionnaires. RC2S uses a personalized approach targeting social cognition and combines pen-and-paper exercises with digital relational simulations using avatars to promote skill transfer. Feasibility and acceptability were examined descriptively and paired-samples *t*-tests assessed exploratory pre–post changes.

**Results:**

All participants who initiated RC2S met the predefined completion criterion (≥50% of sessions), and all completed at least 75% of planned sessions (mean = 20.9). Acceptability was high for cultural adaptation, ease of use, and remote interaction (all>4.1/5), although ratings were lower for the RC2S interface (mean = 3.75/5). Motivation and therapeutic alliance remained high throughout the intervention (MUSIC>5.1;WAI > 4.3). Therapist support, perceived benefits, and remote accessibility were key facilitators. The largest exploratory pre–post changes occurred in therapist-administered goal attainment and functional impact measures. Findings from independently administered measures were more mixed, although a large change was observed for theory of mind.

**Conclusions:**

Findings support the feasibility and acceptability of remotely delivered, culturally adapted RC2S. Exploratory outcomes were mixed, requiring confirmation in a randomized trial with independent, blinded outcome assessment.

## 1 Introduction

Psychotic disorders are associated with social cognitive impairments (Weinreb et al., 2022), which have a substantial impact on everyday functioning and personal recovery (Halverson et al., 2019; Larrauri and Staglin, 2022). Social cognition is defined as the set of mental processes involved in perceiving and interpreting social information in order to guide behavior in social contexts (Corbera et al., 2025; Green et al., 2015). Four domains of social cognition have been identified as central for assessment and intervention in psychotic disorders including emotion processing, social perception and knowledge, theory of mind, and attributional biases (Pinkham et al., 2014).

Evidence regarding the efficacy of pharmacotherapy and brain stimulation for treating social cognitive impairments has been mixed (Vaskinn and Horan, 2020; Yamada et al., 2022). Although findings for social cognitive training (SCT) are encouraging, randomized controlled trials remain scarce, and the evidence is often based on pilot studies with small sample sizes and no control groups (Vaskinn and Horan, 2020; Yeo et al., 2022; Horan and Green, 2019). Current evidence suggests that cognitive remediation targeting social cognition, also referred to as SCT, is associated with significant improvements in emotion recognition, social perception, and theory of mind, with medium to large effect sizes (Yeo et al., 2022; Nijman et al., 2020). However, the effects on attributional biases are small and non-significant (Yeo et al., 2022; Nijman et al., 2020). Similarly, effects on social functioning remain limited, highlighting the need to optimize interventions to enhance the transfer of acquired skills to everyday life (Yeo et al., 2022).

Several factors may explain the limited impact of SCT on functional outcomes, as well as engagement challenges among individuals with psychotic disorders. First, the limited effects on functioning reported in prior studies (Yeo et al., 2022) suggest that ecologically valid approaches are needed to facilitate the transfer of skills to real-life contexts. The use of digital technologies, such as virtual reality (Park et al., 2019) and digital relational simulations with avatars (Peyroux and Franck, 2016), offers a promising avenue for delivering interactive, multimodal environments that facilitate the practice of acquired skills and their transfer to real-world interactions (Oker et al., 2015). These technologies can also incorporate gamification elements, which may support engagement (Cella et al., 2022). Second, given individual differences, a personalized approach is essential to tailor interventions to each individual's needs and to support motivation and engagement (Corbera et al., 2025; Vaskinn and Horan, 2020). Third, access to SCT remains limited and is largely dependent on in-person services (Wykes et al., 2024). Due to the intensive nature of SCT, access can be challenging for individuals who are employed full-time or living far from specialized services. Expanding delivery modalities, including remote formats, may improve accessibility. Although the feasibility and efficacy of remotely delivered cognitive remediation for neurocognition have been examined in recent years (Jagtap et al., 2022; Best et al., 2023; Moura et al., 2019), very few studies have focused on remote SCT (Nahum et al., 2021; Vázquez-Campo et al., 2016).

RC2S (*Remédiation de la Cognition Sociale dans la Schizophrénie*) (Peyroux and Franck, 2014) is an SCT targeting the four core domains of social cognition in psychotic disorders. The program adopts a highly personalized approach based on a case formulation that integrates subjective and objective assessments of social cognition, as well as the development of individualized goals. The program combines pen-and-paper exercises, digital relational simulations with avatars, and homework to support the development, practice, and transfer of strategies to everyday life. Two rigorous case studies conducted in France have supported the feasibility and acceptability of RC2S (Peyroux and Franck, 2016). Additionally, a recent randomized controlled trial comparing RC2S with the neurocognitive remediation program RECOS reported significant improvements in social cognition and social functioning, with greater reductions in hostile attribution biases and overall better functioning at follow-up in the RC2S group (Peyroux et al., 2026). While these findings are encouraging, the well-established influence of culture on social cognition (Weinreb et al., 2022; Hajdúk et al., 2019) must be considered when implementing interventions that rely on verbal and non-verbal cues. This suggests that the original French version of RC2S developed in France requires cultural adaptation for use in different geographical and cultural contexts.

The present pilot study had two main objectives. The first was to translate and culturally adapt the original French version of RC2S for English-Canadian and French-Canadian populations. The second was to evaluate the feasibility, acceptability, engagement, and preliminary outcome changes of remotely delivering these culturally adapted versions using a pre–post design. We hypothesized that remote implementation of RC2S would be both feasible and acceptable, and that participants and therapists would identify facilitators and barriers to its use.

## 2 Methods

### 2.1 Procedure

This study was approved by the Research Ethics Committee of the Montreal West Island Integrated University Health and Social Services Centre (#2022–333, IUSMD-21–34). The study was prospectively registered on ClinicalTrials.gov (NCT05017532; registered August 17, 2021).

The first phase of the study aimed to translate and culturally adapt the original French version of RC2S for English-Canadian and French-Canadian populations. The detailed procedures for translation and cultural adaptation are described in the Materials section of this paper.

The second phase of the study aimed to assess the feasibility, acceptability, and preliminary outcome changes of remotely delivered RC2S using a pre–post quasi-experimental design. This phase included a baseline assessment, a 12-week intervention period, and a post-test assessment. All assessments were administered remotely by a research assistant, with the exception of the ERF-CS and GAS (see Material), which were administered by the treating therapist during the first and final intervention sessions, while the intervention was delivered by a trained therapist. All participants provided informed consent prior to participation. They received financial compensation in the form of gift cards following the baseline and post-test assessments, but not for participation in the intervention.

### 2.2 Participants

The study included two groups of participants: individuals with psychotic disorders and their therapists.

Participants with psychotic disorders were recruited through referrals from healthcare professionals or from a registry of individuals who had participated in previous studies and consented to be re-contacted. They were eligible if they: (1) had a DSM-5 diagnosis of a schizophrenia spectrum disorder or bipolar disorder with psychotic features; (2) were 18 years of age or older; (3) were receiving services at the Douglas Mental Health University Hospital; (4) reported subjective complaints of social cognitive impairment and/or demonstrated objective difficulties in at least one of the four core domains of social cognition at baseline (i.e., performance equal to or below one standard deviation from normative data on selected measures); (5) were considered symptomatically stable and able to use online platforms, as determined by their primary clinician; (6) had access to digital technology, the Internet, and a private space; and (7) were able to provide an emergency contact, as a safety measure for remote intervention delivery. Exclusion criteria included: (1) evidence of an organic cause for cognitive difficulties (e.g., neurological disease, history of brain injury); (2) a diagnosis of intellectual disability or autism spectrum disorder; (3) current hospitalization at the time of recruitment; or (4) inability to speak or read French or English.

Therapists were recruited from the Centre for Personalized Psychological Intervention for Psychosis (Ci3P) clinic and the Comprehensive Research Into Schizophrenia and Psychosis laboratory at the Douglas Institute. They were eligible if they were 18 years of age or older and were psychologists, neuropsychologists, or doctoral-level trainees in psychology or neuropsychology. Participation as research subjects was optional, meaning therapists could deliver the intervention without participating in the research component. However, all therapists agreed to take part as research participants.

### 2.3 Material

#### 2.3.1 Assessment

##### 2.3.1.1 Acceptability and feasibility

Acceptability and feasibility were first assessed using objective indicators, including the number of participants approached and enrolled, the number of dropouts and reasons for withdrawal, the number of sessions completed and missed, and the number of completers (defined in the published protocol as participants who completed at least 50% of the intervention). In addition, during the final therapy session, participants completed a study-specific questionnaire assessing acceptability, usability, safety, perceived impact, and overall satisfaction with the remote intervention. Therapists also completed a questionnaire at the final session to assess their perspectives on and satisfaction with the intervention. All acceptability items in both questionnaires were rated on a Likert scale ranging from 1 (disagree) to 5 (completely agree). Higher scores on these questionnaires were associated with greater satisfaction with the intervention.

No formal quantitative progression criteria for a future randomized controlled trial were specified a priori, beyond the predefined treatment completion criterion of completing at least 50% of the intervention. Recruitment, retention, session attendance, acceptability, and safety were therefore examined descriptively as feasibility indicators rather than evaluated against predetermined success thresholds.

##### 2.3.1.2 Engagement and process-related variables

In-session questionnaires were administered to participants and therapists at sessions 5, 11, and 23 of RC2S to assess motivation and therapeutic alliance. Motivation was measured using the MUSIC® Model of Motivation Inventory—Cognitive Training version (MMI-CT) and its clinician version (Hansen et al., 2019), with scores ranging from 1 to 6, with higher scores indicating greater motivation; participant scores reflect their own motivation toward the intervention, whereas therapist scores reflect their perceptions of the participant's motivation. Therapeutic alliance in the context of remote delivery was assessed using the Working Alliance Inventory—Short Form (WAI) and its therapist version (Tracey and Kokotovic, 1989), with scores ranging from 1 to 7, with higher scores indicating a stronger therapeutic alliance. At the final session, participants completed the eTherapy Attitudes and Process questionnaire (eTAP) (Clough et al., 2019) to assess factors related to engagement in digital mental health interventions. Both participants and therapists also completed a study-specific questionnaire at the final session to identify perceived facilitators and barriers to the remote delivery of the intervention. At the first and final therapy sessions, the therapist administered the Goal Attainment Scale (Turner-Stokes, 2009), which operationalizes individualized treatment goals and provides quantitative ratings of goal attainment at the end of the intervention. Given its individualized and collaboratively defined nature, the GAS was considered an indicator of progress toward treatment goals and engagement in the therapeutic process rather than an independent measure of intervention efficacy. Because the GAS was embedded within the intervention and administered by the treating therapist, the assessments were not blinded to intervention exposure or assessment timepoint.

##### 2.3.1.3 Preliminary outcome changes following remote RC2S

The following measures were administered at baseline and post-test. Social cognition was assessed using the Combined Stories Test (Achim et al., 2012; Thibaudeau et al., 2018; Achim et al., 2025) for theory of mind, the Social Knowledge Test (Achim et al., 2012; Thibaudeau et al., 2018; Achim et al., 2025) for social knowledge, the Penn Emotion Recognition Test (Kohler et al., 2003) for emotion recognition and the Internal, Personal and Situational Attributions Questionnaire (IPSAQ) (Kinderman and Bentall, 1996) for attributional biases. Cognitive biases were assessed using the Davos Assessment of Cognitive Biases Scale (DACOBS) (van der Gaag et al., 2013). Clinical symptoms were evaluated with the Positive and Negative Syndrome Scale (PANSS-6) (Østergaard et al., 2016) for psychotic symptoms, the Social Interaction Anxiety Scale (SIAS) (Mattick and Clarke, 1998) for social anxiety and the Patient Health Questionnaire (PHQ-9) (Kroenke et al., 2001) for depressive symptoms. The First-episode social functioning scale (FESFS) (Lecomte et al., 2014) was used to assess functioning while recovery was evaluated using the Questionnaire about the process of recovery (QPR) (Neil et al., 2009).

At the first and final therapy sessions, the therapist administered the Échelle de Répercussions Fonctionnelles des troubles de la Cognition Sociale (Scale of Functional Impact of Social Cognition Disorders; ERF-CS) (Gaudelus et al., 2018), a semi-structured interview designed to assess the functional impact of social cognitive impairments in daily life across the domains of emotion processing, social perception, theory of mind, and attributional biases.

#### 2.3.2 Intervention

RC2S (Peyroux and Franck, 2016; Peyroux and Franck, 2014) is an SCT program focused on the development of skills and strategies. The intended intervention dose consists of 24 individual one-hour sessions with a therapist, delivered twice weekly over 12 weeks. The intervention was delivered remotely using Zoom for Healthcare. RC2S targets four core domains of social cognition: emotion processing, social perception, theory of mind, and attributional biases.

**Fig. 1:**
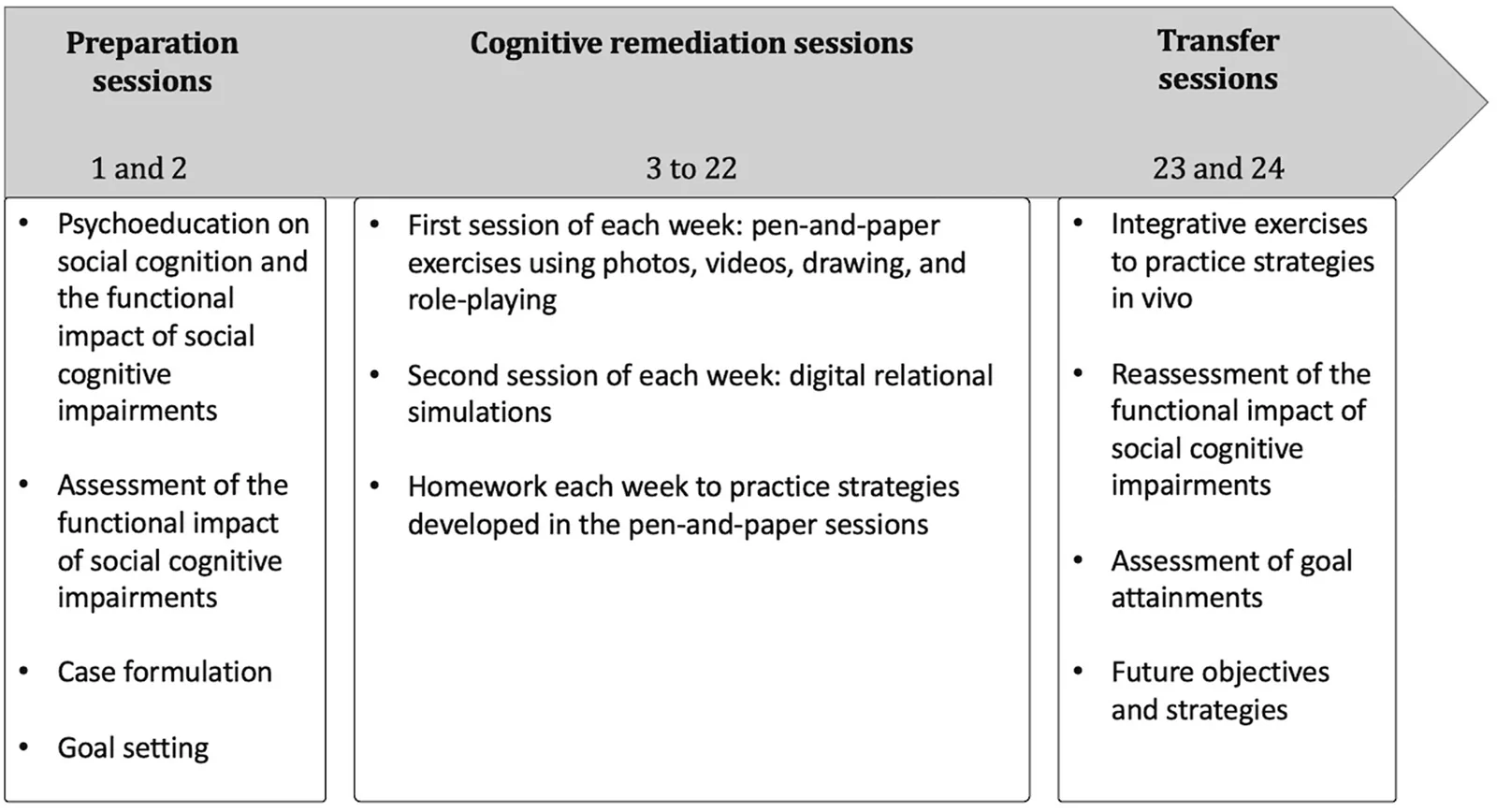
Outline of RC2S sessions.

The structure of the sessions is presented in Fig. 1. The first two sessions are dedicated to preparation, allowing the therapist to become familiar with the participant and to tailor the intervention. Participants receive psychoeducation about social cognition, and a semi-structured interview using the ERF-CS (Gaudelus et al., 2018) is conducted to obtain a subjective assessment of social cognitive abilities and their functional impact, complementing the objective baseline assessment. During these sessions, participants formulate one to three goals in accordance with the COG-SMART principles (Wykes et al., 2016), using the Goal Attainment Scale (Turner-Stokes, 2009) to define specific anchors indicating whether goals are achieved, partially achieved, or exceeded after the intervention. Based on this case formulation, the therapist determines the number of sessions allocated to each social cognitive domain, as well as the specific cognitive remediation strategies to be used, according to the participants' profile.

**Fig. 2:**
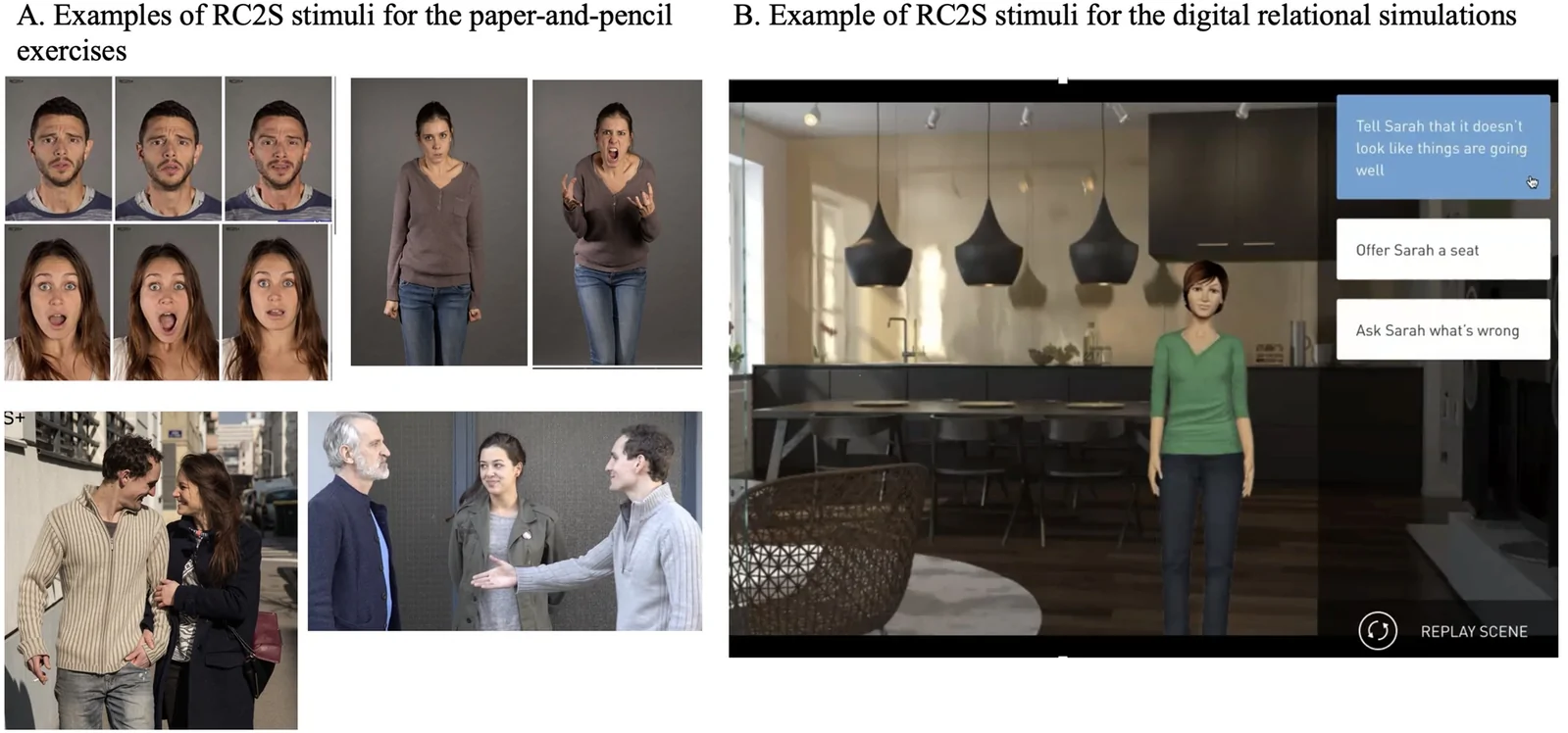
Illustration of RC2S stimuli.

Sessions 3 to 22 are dedicated to cognitive remediation. In the first session of each week, participants complete pen-and-paper exercises based on photographs and videos available on the RC2S platform (see Fig. 2A for examples). These exercises incorporate evidence-based cognitive remediation techniques and strategies. In the second weekly session, integrative digital relational simulations involving avatars (see Fig. 2B for an example) are used to practice the strategies developed during the last pen-and-paper session. In these simulations, participants assist a character named Tom in navigating increasingly complex social interactions, with scenarios evolving dynamically based on participants' choices. Homework is assigned after the first weekly session to facilitate the application of learned strategies in daily life.

The final two sessions focus on transfer, incorporating integrative exercises such as personalized role-plays. These sessions also include a reassessment of the functional impact of social cognitive impairments using the ERF-CS, as well as a review of goal attainment. Future objectives related to social cognition and strategies to achieve them are also discussed.

The intervention was delivered by psychologists, neuropsychologists, and doctoral-level trainees in psychology or neuropsychology. Therapists received an initial one-day training led by ET, an experienced cognitive remediation therapist trained by the developer of RC2S (EP). They also participated in weekly group supervision sessions to discuss ongoing cases and ensure treatment fidelity.

To translate and culturally adapt the original French version of RC2S for English-Canadian and French-Canadian populations, a professional translator first translated the pen-and-paper exercises and digital relational simulations into English. This initial translation was reviewed and refined by the bilingual research team to ensure fidelity to the original content. In parallel, all intervention materials and scenarios were reviewed for their cultural and contextual relevance to Canadian users. Because most of the everyday social situations and contextual elements of the original French version were considered readily transferable to the Canadian context, only minor cultural adaptations were required. These included modifying character names, references to educational levels, names of public places, and culturally specific expressions or idioms. For the French-Canadian version, two team members reviewed all materials and adapted language and expressions to reflect French-Canadian usage, with a third member consulted in cases of disagreement. No changes to the core therapeutic content, targeted social cognitive processes, or structure of the intervention were considered necessary. Following this process, professional actors and a recording studio were engaged to produce audio recordings in both English-Canadian and French-Canadian. The research team then integrated the new audio files into the digital relational simulations. A pilot test was conducted to ensure proper integration and functioning of all recordings.

#### 2.3.3 Statistical analyses

Descriptive statistics were first used to summarize the sociodemographic and clinical characteristics of participants and therapists. For acceptability and feasibility, descriptive statistics were used to summarize objective indicators, as well as the mean scores of the acceptability questionnaires.

For engagement and process-related variables, descriptive statistics were used to summarize scores from the MMI-CT, the MMI-CT-C, the WAI, the WAI-T, the eTAP, the GAS as well as the study-specific questionnaires. Responses to open-ended questions regarding perceived facilitators and barriers to engagement were categorized, and their prevalence was quantified using descriptive statistics.

To explore pre–post changes, paired-samples *t*-tests were used to assess within-participant change. Analyses were conducted using available paired observations for each outcome. Missing observations were not imputed; consequently, sample sizes varied across outcomes according to the availability of paired baseline and post-test data. For the ERF-CS and the Goal Attainment Scale (GAS) paired-samples *t*-tests similarly compared scores obtained during the first and final therapy sessions. Effect sizes were expressed as Cohen's dz. with 95% confidence intervals. Statistical significance was set at *p* < 0.05, while *p*-values between 0.05 and 0.10 were described as marginally significant for descriptive purposes. Given the pilot and hypothesis-generating nature of the study, no adjustment for multiple comparisons was applied. All *p*-values are therefore unadjusted and should be interpreted as exploratory rather than as confirmatory tests of intervention efficacy. JASP 0.18.1 was used for the analyses.

## 3 Results

### 3.1 Feasibility and acceptability

Seven therapists were recruited, including six women and one man, with a mean age of 28.3 years (SD = 2.5). Two therapists were licensed neuropsychologists, while the remaining five were doctoral-level students in psychology or neuropsychology. Their sociodemographic characteristics are presented in Table 1, alongside those of participants with psychotic disorders. Although we initially planned to recruit five therapists (Thibaudeau et al., 2024), two additional therapists were recruited due to staff turnover during the project.

**Table 1:** Sociodemographic and clinical information for participants with psychotic disorders and therapists.

	Psychotic disorders (*N* = 14)			Therapists (*N* = 7)		
	Mean	SD	%	Mean	SD	%
Gender (%Women)			42.9			85.7
Age	37.8	10.0		28.3	2.5	
Education	12.9	2.9		18.3	1.9	
Ethnicity	White Black Arab Asian		71.4% 21.5% 7.1% 0%			85.7% 0% 0% 14.3%
Duration of illness	12.5	7.1				
Chlorpromazine equivalent	424.6	404.6				
Primary diagnosis	Schizophrenia spectrum disorder Bipolar with psychotic features		64.3% 35.7%			

**Fig. 3:**
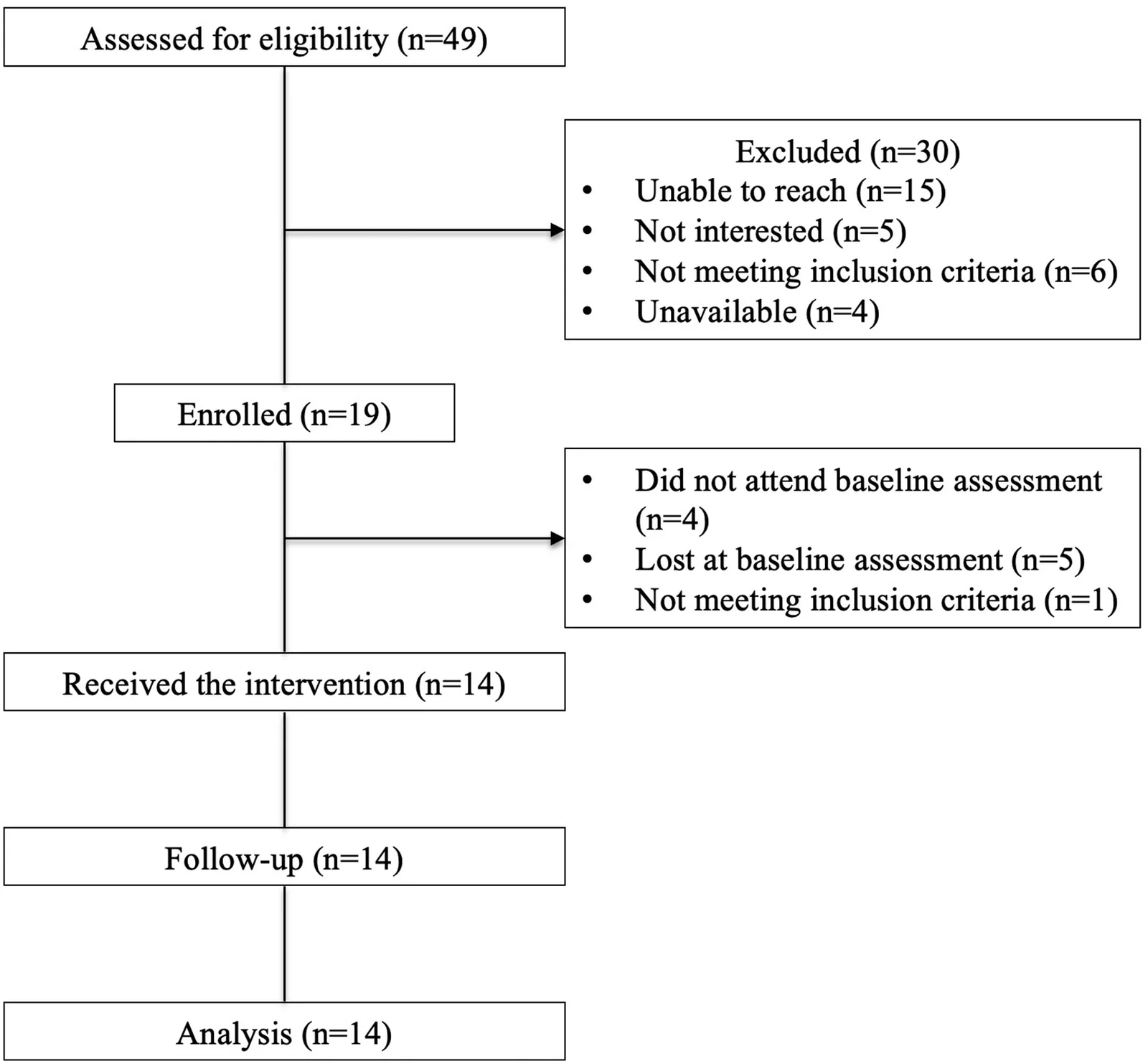
Participant flow diagram.

The participant flow diagram for participants with a psychotic disorder is presented in Fig. 3. A total of 49 prospective participants with a psychotic disorder were referred or approached for participation, of whom 19 were enrolled in the study. This corresponded to an enrollment yield of 38.8% (19/49) among individuals referred or approached and an overall approach-to-completion throughput of 28.6% (14/49). Among enrolled participants, 73.7% (14/19) completed the baseline and post-intervention assessments and met the predefined intervention completion criterion, although one of the five non-completers was subsequently found to be ineligible. Their sociodemographic and clinical characteristics are presented in Table 1. The therapy was delivered in French for 8 participants, and in English for 6 participants.

Two modifications to the published protocol (Thibaudeau et al., 2024) regarding participants with psychotic disorders were made. First, although we initially planned to recruit 20 participants, recruitment was terminated early due to staff turnover during the project. Second, while the original protocol specified the inclusion of participants with schizophrenia spectrum disorders only, the criteria were expanded to include individuals with bipolar disorder with psychotic features. Given the substantial overlap in social cognitive impairments and the presence of psychotic symptoms across these conditions, a transdiagnostic approach was considered appropriate, allowing the intervention to be offered to individuals receiving care in units specialized in psychotic disorders. In addition, a consideration concerned the definition of intervention completion and adherence. The published protocol defined treatment completion as completion of at least 50% of the intervention. In the present manuscript, we also elected to report a more stringent threshold of 75% of planned sessions as a complementary indicator of intervention adherence. Accordingly, both thresholds are reported: the predefined ≥50% criterion is retained as the formal definition of treatment completion, while completion of ≥75% of sessions is presented as a more stringent descriptive indicator of adherence.

The intended intervention dose was 24 one-hour sessions delivered twice weekly over 12 weeks. Participants completed an average of 20.9 sessions (SD = 2.8), corresponding to 87.1% of the planned intervention dose. All 14 participants who initiated the intervention (100%) met the predefined treatment completion criterion of completing at least 50% of the intervention. Notably, all participants also completed at least 75% of the planned sessions. One participant ended the intervention after completing 19 sessions following a change in employment to a position with atypical hours, which prevented further scheduling of sessions. On average, participants missed 1.7 sessions (SD = 2.5) for various reasons, including forgetting scheduled sessions, illness or fatigue, and scheduling conflicts (e.g., medical appointments or work commitments).

Participants' acceptability ratings indicated high levels of satisfaction with the remote therapeutic relationship (4.4/5), cultural adaptation of the program (4.1/5), ease of use (4.3/5), and overall satisfaction with the intervention (4.7/5). Participants also reported feeling safe during the remote intervention (4.6/5). However, ratings for the program interface were comparatively lower (3.8/5).

Similarly, therapists reported high acceptability scores for the remote therapeutic relationship with participants (4.7/5), cultural adaptation (4.5/5), ease of use (4.3/5), and overall satisfaction (4.2/5). Their responses also indicated that they perceived several benefits of using RC2S in their current or future clinical practice (4.4/5). As observed among participants, therapists rated the visual interface of RC2S lower (3.7/5) than other aspects of acceptability.

### 3.2 Engagement and process-related variables

**Fig. 4:**
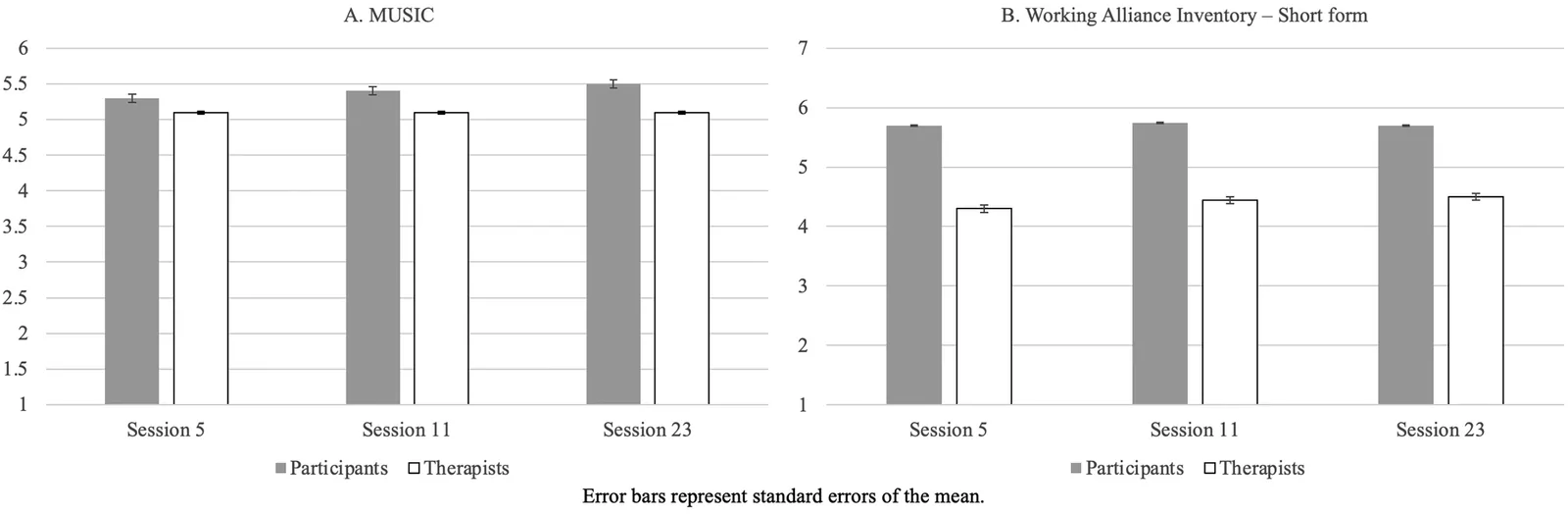
Intrasession questionnaires scores.

Motivation toward the remote intervention showed high and stable mean scores across sessions 5, 11, and 23 for both participants and therapists (see Fig. 4A). Similarly, therapeutic alliance scores were high and stable across these sessions for participants, while therapists reported moderate to high levels of therapeutic alliance (see Fig. 4B).

Reasons for engaging in remote RC2S were assessed using the eTAP. All participants reported engaging in the intervention to improve their understanding of their difficulties and how to overcome them (100%). Many participants also indicated a desire to learn specific skills (78.6%) or to receive support with new projects (64.3%). Additionally, some participants reported engaging in the intervention to receive support from mental health service providers (42.9%) or to contribute to research and feel helpful (35.7%).

Participants and therapists identified several facilitators of engagement with the intervention, including personal motivation and involvement, the presence of an active and empathetic therapist, perceived benefits of the intervention, insight into one's difficulties, and the flexibility afforded by remote delivery. Both groups also reported potential barriers to engagement, including low motivation or personal involvement, difficulty discussing personal difficulties, full-time work or academic commitments, and limited access to a device or reliable internet connection.

As an individualized indicator of progress within the therapeutic process, goal attainment showed a statistically significant increase from the first to the final session, t(12) = −6.19, *p* < 0.001, dz. = 1.72.

### 3.3 Preliminary outcome changes following remote RC2S

The results of the paired-samples *t*-tests comparing baseline and pos*t*-test scores are presented in Table 2.

**Table 2:** Scores and changes in the cognitive, clinical and psychosocial measures following RC2S.

		Baseline	Post-test	t-test	Cohen's dz	95% CI for Cohen's dz	
						Lower	Upper
Goal attainment		−1.52 (0.52)	0.38 (0.89)	t(12) = −6.187, *p* < 0.001⁎	−1.72	−2.57	−0.83

Social cognition							
	ER-40	33.9 (3.4)	33.6 (3.7)	t(13) = 0.248, *p* = 0.808	0.07	−0.46	0.59
	SKT	10.5 (2.2)	11.4 (1.7)	t(13) = −1.608, *p* = 0.132	−0.43	−0.97	0.13
	COST	39.0 (5.7)	43.5 (3.8)	t(13) = −3.798, *p* = 0.002⁎	−1.01	−1.65	−0.35
	IPSAQ – Externalizing bias	1.6 (4.8)	1.4 (5.3)	t(13) = 0.140, *p* = 0.891	0.04	−0.49	0.56
	IPSAQ – Personalising bias	0.3 (0.2)	0.4 (0.2)	t(13) = −1.275, *p* = 0.225	−0.34	−0.87	0.21

Functional impact of social cognition (ERF-CS)							
	Emotion processing	25.1 (9.6)	16.5 (8.3)	t(10) = 5.216, *p* < 0.001⁎	1.57	0.66	2.46
	Social perception	13.4 (8.5)	9.2 (6.7)	t(10) = 2.114, *p* = 0.061+	0.64	−0.03	1.28
	Theory of mind	16.2 (10.8)	9.2 (5.1)	t(10) = 2.728, *p* = 0.021⁎	0.82	0.118	1.49
	Attributional bias	14.2 (8.9)	10.2 (8.2)	t(10) = 2.187, *p* = 0.054+	0.66	−0.01	1.30
	Overall social cognition	67.3 (29.7)	45.1 (19.9)	t(10) = 4.112, *p* = 0.002⁎	1.24	0.43	2.02

First-episode social functioning scale							
	Autonomy	13.0 (1.7)	13.4 (1.5)	t(13) = −0.806, *p* = 0.435	−0.22	−0.74	0.32
	Social interactions	12.1 (2.0)	13.0 (2.5)	t(13) = −1.531, *p* = 0.150	−0.41	−0.95	0.14
	Social activities	14.9 (3.3)	16.6 (3.0)	t(13) = −2.033, *p* = 0.063+	−0.54	−1.09	0.03
	Intimacy	14.9 (3.0)	14.9 (3.2)	t(13) = 0.000, *p* = 0.999	0.00	−0.52	0.52
	Family	9.1 (1.6)	7.9 (2.8)	t(13) = 1.578, *p* = 0.139	0.42	−0.13	0.96
	Relationships at work	9.5 (1.2)	9.1 (2.0)	t(3) = 1.095, *p* = 0.353	0.55	−1.58	0.55

Cognitive biases (DACOBS)							
	Jumping to conclusions	23.8 (7.8)	21.9 (3.6)	t(13) = 0.823, *p* = 0.425	0.22	−0.31	0.75
	Inflexibility	19.8 (9.7)	15.8 (5.6)	t(13) = 1.913, *p* = 0.078+	0.51	−0.06	1.06
	Threat	27.1 (7.6)	24.6 (5.1)	t(13) = 0.998, *p* = 0.336	0.27	−0.27	0.79
	External bias	21.1 (8.1)	18.7 (6.7)	t(13) = 0.818, *p* = 0.428	0.22	−0.32	0.75
	Social cognitive problem	23.9 (8.1)	19.9 (6.2)	t(13) = 2.307, *p* = 0.038⁎	0.62	0.03	1.18
	Subjective complaint	27.4 (7.7)	22.1 (7.4)	t(13) = 3.315, *p* = 0.006⁎	0.89	0.25	1.50
	Safety	17.9 (10.4)	14.8 (7.9)	t(13) = 1.246, *p* = 0.235	0.33	−0.21	0.87

Clinical symptoms							
	SIAS	35.7 (14.8)	37.6 (9.5)	t(13) = −0.649, *p* = 0.528	−0.17	−0.70	0.36
	PHQ-9	10.2 (5.2)	9.1 (4.2)	t(13) = 0.679, *p* = 0.509	0.18	−0.35	0.71
	PANSS-6 – total	13.1 (4.4)	11.5 (5.1)	t(13) = 1.504, *p* = 0.157	0.40	−0.15	0.94
	PANSS-6 – positive	6.4 (3.3)	5.6 (3.7)	t(13) = 1.249, *p* = 0.234	0.33	−0.21	0.87
	PANSS-6 – negative	6.7 (2.9)	5.9 (2.6)	t(13) = 1.115, *p* = 0.285	0.30	−0.24	0.83

Recovery	QPR	56.8 (11.3)	60.1 (10.8)	t(13) = −1.032, *p* = 0.321	−0.28	−0.81	0.26

All *p*-values are unadjusted for multiple comparisons and are presented for exploratory purposes.

Effect sizes are reported as Cohen's dz. for paired observations, with the sign corresponding to the baseline minus post-test contrast.

ER-40: Penn Emotion Recognition Task; SKT: Social Knowledge Test; COST: Combined Stories Test; IPSAQ: Internal, Personal and Situational Attributions Questionnaire; ERF-CS: Échelle de repercussions fonctionnelles des troubles de la cognition sociale; QPR: Questionnaire about the process of recovery; DACOBS: Davos Assessment of Cognitive Biases Scale; SIAS: Social Interaction Anxiety Scale; PHQ-9: Patient Health Questionnaire; PANSS-6: Positive and Negative Syndrome Scale.

⁎ Statistically significant change (*p* < 0.05).

+ Marginally significant change.

Among social cognition measures administered independently of the treating therapist, a statistically significant pre-post change with a large effect size was observed for theory of mind on the COST t(13) = −3.80, *p* = 0.002, dz. = 1.01, whereas no statistically significant pre–post changes were observed on the ER-40, SKT, or IPSAQ.

For the therapist-administered ERF-CS, statistically significant reductions in ratings of the functional impact of social cognitive difficulties were observed for overall social cognition t(10) = 4.11, *p* = 0.002, dz. = 1.24, as well as for emotion processing, t(10) = 5.22, *p* < 0.001, dz. = 1.57, and theory of mind, t(10) = 2.73, *p* = 0.021, dz. = 0.82. Marginally significant pre–post changes were observed for social perception, t(10) = 2.11, *p* = 0.061, dz. = 0.64, and attributional biases, t(10) = 2.19, *p* = 0.054, dz. = 0.66.

Regarding functioning, marginally significant pre–post change was observed for the social activities scale t(13) = 2.03, *p* = 0.063, dz. = 0.54.

For cognitive biases, statistically significant pre-post changes were observed for the DACOBS social cognitive problems t(13) = 2.31, *p* = 0.038, dz. = 0.62, and subjective complaints scales t(13) = 3.32, *p* = 0.006, dz. = 0.89. A marginally significant pre-post change was also observed for inflexibility bias t(13) = 1.91, *p* = 0.078, dz. = 0.51.

No significant changes were observed for clinical symptoms or recovery.

## 4 Discussion

This study examined the feasibility, acceptability, engagement, and exploratory pre-post changes associated with the remote delivery of two culturally adapted versions of RC2S to individuals with psychotic disorders. Acceptability and engagement were high, and feasibility indicators were encouraging, although recruitment and overall study throughput were more modest than retention. Exploratory outcomes varied by measurement method: the largest changes occurred on therapist-administered goal attainment and functional impact measures, while independently administered measures were more mixed, with a large change observed on the COST measure of theory of mind.

### 4.1 Feasibility and acceptability of remote and culturally adapted RC2S

Of the 49 individuals referred or approached, 19 (38.8%) enrolled and 14 (28.6%) completed the intervention and post-test assessment. Four enrolled participants did not attend baseline and one was subsequently found ineligible. Thus, a future trial should account for loss between referral and intervention initiation when setting recruitment targets.

Although attrition is a recognized challenge in psychosocial interventions for psychotic disorders (Wojtalik et al., 2023), retention and adherence after remote RC2S initiation were encouraging. All participants met the predefined ≥50% completion criterion and completed at least 75% of planned sessions. The original in-person RC2S trial reported an attrition rate of 16% (Peyroux et al., 2026); although designs differ, the present findings suggest that remote delivery is compatible with good intervention retention and engagement.

Engagement was also reflected in sustained motivation. RC2S is highly personalized, integrating participants' goals, cognitive profiles, and functional difficulties with support from an active and trained therapist, a component associated with better cognitive remediation outcomes (Wykes et al., 2024; Vita et al., 2021). All participants progressed toward individualized goals; however, because GAS goals were collaboratively defined and rated by the treating therapist, these findings are best interpreted as a process indicator rather than evidence of efficacy. Progress toward personally meaningful goals may nevertheless contribute to the acceptability and clinical relevance of personalized cognitive remediation (Wykes et al., 2024).

Satisfaction with the cultural adaptations was also high, although the predominantly White sample limits conclusions across cultural groups. Interestingly, given the active role of therapists in RC2S and the high level of personalization within the program, these features may contribute to enhancing cultural responsiveness.

RC2S may further support engagement and skill transfer through progressively complex stimuli and avatar-based simulations that provide multimodal practice beyond static tasks (Oker et al., 2015; Wykes et al., 2024). These simulations emphasize interaction dynamics (Froese et al., 2014), provide a more ecologically valid representation of the rapid and multimodal processes involved in everyday social interactions (Dilgul et al., 2020; Salahuddin et al., 2024; Schroeder et al., 2022), and allow supported practice in a controlled setting (Peyroux and Franck, 2014). Further, given the high prevalence of negative symptoms in psychotic disorders (e.g. avolition, anhedonia) (Galderisi et al., 2018), it is essential to develop cognitive remediation interventions that are not only evidence-based but also incorporate gamification to address these challenges (Oker et al., 2015; Cella et al., 2022).

Participants and therapists nevertheless rated the visual aspects of the avatars and environments less favorably. While these limitations did not appear to negatively affect motivation, engagement, or the overall acceptability of the remote intervention, future iterations of the program should aim to enhance these visual features. Additional improvements may include expanding the range of scenarios or leveraging artificial intelligence to develop adaptive simulations that adjust in real time to participants' responses, thereby further enhancing personalization, engagement, and ecological validity (Sharma et al., 2024).

### 4.2 Remote administration of RC2S

Remote delivery of RC2S had not previously been examined. To our knowledge, only two prior studies have evaluated remotely delivered SCT (Nahum et al., 2021; Vázquez-Campo et al., 2016), neither with the same level of active therapist involvement. This is relevant because trained, actively engaged therapists are associated with stronger cognitive remediation effects (Vita et al., 2021). In the present study, therapeutic alliance remained high, and both remote delivery and therapist support were identified as facilitators. Remote delivery may reduce barriers to cognitive remediation access (Wykes et al., 2024; Calzavara-Pinton et al., 2024), particularly for individuals living far from specialized services or balancing employment, education, or family responsibilities (Calzavara-Pinton et al., 2024). RC2S is well suited to this format because its platform supports real-time therapist-participant collaboration. Digital mental health interventions are also generally acceptable to individuals with psychotic disorders, among whom smartphone ownership and overall access to technology are high (Abdel-Baki et al., 2017; Bucci et al., 2018; Eisner et al., 2023). However, some participants and therapists preferred in-person care, suggesting that flexible or preference-based delivery may optimize engagement.

### 4.3 Preliminary outcome changes following remote RC2S

Exploratory outcomes differed across measurement approaches. The largest changes were observed on the GAS and ERF-CS, both administered by non-blinded treating therapists. Among independently administered social cognitive measures, a large pre–post change was observed on the COST, whereas the ER-40, SKT, and IPSAQ showed no statistically significant changes. Social activities showed only a marginal change, while selected self-reported cognitive bias measures changed significantly. Given the small sample, uncontrolled design, and absence of correction for multiple comparisons, these outcomes should be considered hypothesis-generating rather than evidence of efficacy.

Further, the discrepancy between the ER-40 and the emotion processing scale of the ERF-CS may partly reflect their different targets: the ER-40 assesses recognition of static facial expressions, whereas the therapist-administered ERF-CS assesses the everyday functional impact of social cognitive difficulties. Lower ERF-CS scores could therefore reflect better management or compensatory strategy use rather than improved facial emotion recognition, although expectancy or observer effects cannot be excluded. Differences across social cognitive domains may also reflect responsiveness to training and intervention content. The COST may align more closely with RC2S's contextually rich exercises requiring interpretation of intentions, beliefs, and social cues, whereas the ER-40 assesses a more circumscribed skill. The lack of change on the IPSAQ is also consistent with evidence that attributional biases may be less responsive to SCT (Yeo et al., 2022; Nijman et al., 2020). These interpretations remain tentative because RC2S is individualized and the amount of training devoted to each domain varied across participants. Although discrepancies between perceived and objectively assessed abilities may also relate to introspective accuracy, the ER-40 provides no trial-by-trial feedback and this study did not assess confidence or self-evaluation accuracy. Similarly, the absence of changes in jumping-to-conclusions and external attribution biases suggests selective rather than generalized change but cannot establish impaired introspective accuracy.

### 4.4 Limitations

The main limitations are the small sample and absence of a control group. Observed pre–post changes cannot necessarily be attributed to RC2S and may reflect practice or retest effects, regression to the mean, expectancy or other nonspecific effects, natural variation, or chance. Multiple exploratory outcomes were tested without multiplicity adjustment, increasing Type I error risk. A randomized controlled trial is therefore needed to evaluate efficacy and should also compare remote and in-person delivery.

No formal quantitative progression criteria were specified a priori beyond the ≥50% treatment completion criterion. Feasibility findings are therefore descriptive rather than evaluations against prespecified benchmarks. To guide progression to a future RCT, we propose a set of pragmatic feasibility criteria covering recruitment, retention, intervention adherence, acceptability, and safety. Specifically, the future trial would be considered to have met its feasibility targets if at least 75% of the planned sample were recruited within the prespecified recruitment period, with site-specific recruitment expectations defined according to the number of participating sites; at least 75% of randomized participants were retained through post-intervention assessment; at least 75% of participants assigned to RC2S completed at least 75% of planned sessions; mean acceptability ratings were ≥ 4/5; and no serious intervention-related adverse events occurred. These thresholds are intended as pragmatic progression criteria informed by the present pilot findings and will be finalized prospectively in the future trial protocol. A qualitative component would also provide a richer understanding of participant and therapist experiences and is being incorporated into ongoing work.

Outcome assessment presents an additional limitation. The GAS and ERF-CS were administered by treating therapists who were not blinded to timepoint or intervention exposure, and most therapists were research team members, creating potential expectancy, observer, and allegiance effects. Future trials should separate treatment delivery from outcome assessment and use independent, blinded assessors.

Finally, although remote delivery reduces travel and scheduling barriers, RC2S remains intensive, requiring up to 24 individual therapist-led sessions. Differences in availability and reimbursement of such care across healthcare systems may limit scalability and generalizability. Future implementation studies should examine delivery resources and costs and whether adaptations can preserve personalized therapist support while improving scalability.

## 5 Conclusion

The findings provide preliminary support for the feasibility of remotely delivered, culturally adapted RC2S and indicate high acceptability and engagement. Exploratory outcomes were mixed across measurement methods, with the largest changes observed in non-blinded therapist-administered measures and a large change on one independently administered theory-of-mind measure. These findings require confirmation in a randomized trial using independent, blinded outcome assessment.

## CRediT authorship contribution statement

**Elisabeth Thibaudeau:** Writing – original draft, Supervision, Project administration, Methodology, Investigation, Funding acquisition, Formal analysis, Data curation, Conceptualization. **Elodie Peyroux:** Writing – review & editing, Validation, Supervision, Software. **Nicolas Franck:** Writing – review & editing, Validation, Software. **Zelda Prost:** Writing – review & editing, Validation, Supervision. **Martin Lepage:** Writing – review & editing, Resources, Methodology, Funding acquisition, Conceptualization.

## Funding

This work was supported by Healthy Brain Healthy Lives funding; M.L was supported by a James McGill Professorship; ET received a postdoctoral fellowship funding from CIHR [#171198, 2020]. The funding sources were not involved in the study design, the collection, analysis and interpretation of data, the writing of the report nor in the decision to submit the article for publication.

## Declaration of competing interest

The authors declare that they have no known competing financial interests or personal relationships that could have appeared to influence the work reported in this paper.

## Data Availability

Data are available upon request.

## References

[bb0005] Abdel-Baki A. (2017). Understanding access and use of technology among youth with first-episode psychosis to inform the development of technology-enabled therapeutic interventions. Early Interv. Psychiatry.

[bb0010] Achim A.M. (2012). Mentalizing in first-episode psychosis. Psychiatry Res..

[bb0015] Achim A.M. (2025). Psychometric properties of the social knowledge test (SKT) and the combined stories test (COST) in people with a schizophrenia spectrum disorder. Schizophr. Res. Cogn..

[bb0020] Best M.W. (2023). Efficacy of remotely delivered evidence-based psychosocial treatments for schizophrenia-spectrum disorders: a series of systematic reviews and meta-analyses. Schizophr. Bull..

[bb0025] Bucci S. (2018). Early psychosis service user views on digital technology: qualitative analysis. JMIR mental health.

[bb0030] Calzavara-Pinton I. (2024). Treatment of cognitive impairment associated with schizophrenia spectrum disorders: new evidence, challenges, and future perspectives. Brain Sci..

[bb0035] Cella M. (2022). Virtual reality therapy for the negative symptoms of schizophrenia (V-NeST): a pilot randomised feasibility trial. Schizophr. Res..

[bb0040] Clough B.A. (2019). Development of the eTAP: a brief measure of attitudes and process in e-interventions for mental health. Internet Interv..

[bb0045] Corbera S. (2025). International perspective on social cognition in schizophrenia: current stage and the next steps. Eur. Psychiatry.

[bb0050] Dilgul M. (2020). Cognitive behavioural therapy in virtual reality treatments across mental health conditions: a systematic review. Consortium Psychiatricum.

[bb0055] Eisner E., Berry N., Bucci S. (2023). Digital tools to support mental health: a survey study in psychosis. BMC Psychiatry.

[bb0060] Froese T., Iizuka H., Ikegami T. (2014). Embodied social interaction constitutes social cognition in pairs of humans: a minimalist virtual reality experiment. Sci. Rep..

[bb0065] Galderisi S. (2018). Negative symptoms of schizophrenia: new developments and unanswered research questions. Lancet Psychiatry.

[bb0070] Gaudelus B. (2018). L’évaluation des répercussions fonctionnelles des altérations de la cognition sociale favorise-t-elle l’engagement dans les soins des personnes ayant des troubles psychotiques?. Ann. Med. Psychol. Rev. Psychiatr..

[bb0075] Green M.F., Horan W.P., Lee J. (2015). Social cognition in schizophrenia. Nat. Rev. Neurosci..

[bb0080] Hajdúk M. (2019). How to move forward in social cognition research? Put it into an international perspective. Schizophr. Res..

[bb0085] Halverson T.F. (2019). Pathways to functional outcomes in schizophrenia spectrum disorders: Meta-analysis of social cognitive and neurocognitive predictors. Neurosci. Biobehav. Rev..

[bb0090] Hansen M.C. (2019). Validation of the MUSIC model of motivation inventory for use with cognitive training for schizophrenia spectrum disorders: a multinational study. Schizophr. Res..

[bb0095] Horan W.P., Green M.F. (2019). Treatment of social cognition in schizophrenia: current status and future directions. Schizophr. Res..

[bb0100] Jagtap S. (2022). Can cognitive remediation therapy be delivered remotely? A review examining feasibility and acceptability of remote interventions. Schizophr. Res. Cogn..

[bb0105] Kinderman P., Bentall R.P. (1996). A new measure of causal locus: the internal, personal and situational attributions questionnaire. Personal. Individ. Differ..

[bb0110] Kohler C.G. (2003). Facial emotion recognition in schizophrenia: intensity effects and error pattern. Am. J. Psychiatry.

[bb0115] Kroenke K., Spitzer R.L., Williams J.B. (2001). The PHQ-9: validity of a brief depression severity measure. J. Gen. Intern. Med..

[bb0120] Larrauri C.A., Staglin B. (2022). Leading science with lived experience. Schizophr. Bull..

[bb0125] Lecomte T. (2014). Development and preliminary validation of the first episode social functioning scale for early psychosis. Psychiatry Res..

[bb0130] Mattick R.P., Clarke J.C. (1998). Development and validation of measures of social phobia scrutiny fear and social interaction anxiety. Behav. Res. Ther..

[bb0135] Moura B.M. (2019). Facilitating the delivery of cognitive remediation in first-episode psychosis: pilot study of a home-delivered web-based intervention. J. Nerv. Ment. Dis..

[bb0140] Nahum M. (2021). Online social cognition training in schizophrenia: a double-blind, randomized, controlled multi-site clinical trial. Schizophr. Bull..

[bb0145] Neil S.T. (2009). The questionnaire about the process of recovery (QPR): a measurement tool developed in collaboration with service users. Psychosis.

[bb0150] Nijman S.A. (2020). Social cognition training for people with a psychotic disorder: a network meta-analysis. Schizophr. Bull..

[bb0155] Oker A. (2015). How and why affective and reactive virtual agents will bring new insights on social cognitive disorders in schizophrenia? An illustration with a virtual card game paradigm. Front. Hum. Neurosci..

[bb0160] Østergaard S.D. (2016). PANSS-6: a brief rating scale for the measurement of severity in schizophrenia. Acta Psychiatr. Scand..

[bb0165] Park M.J. (2019). A literature overview of virtual reality (VR) in treatment of psychiatric disorders: recent advances and limitations. Front. Psychol..

[bb0170] Peyroux E., Franck N. (2016). Improving social cognition in people with schizophrenia with RC2S: two single-case studies. Front. Psychol..

[bb0175] Peyroux E. (2026). Is the Cognitive Remediation of Social Cognition (RC2S) program useful to improve social cognition and functional outcomes in people with schizophrenia? A randomized controlled trial. Psychiatr. Rehabil. J..

[bb0180] Peyroux E., Franck N. (2014). RC2S: a cognitive remediation program to improve social cognition in schizophrenia and related disorders. Front. Hum. Neurosci..

[bb0185] Pinkham A.E. (2014). The social cognition psychometric evaluation study: results of the expert survey and RAND panel. Schizophr. Bull..

[bb0190] Salahuddin N.H. (2024). Psychological and psychosocial interventions for treatment-resistant schizophrenia: a systematic review and network meta-analysis. Lancet Psychiatry.

[bb0195] Schroeder A.H. (2022). Feasibility and efficacy of virtual reality interventions to improve psychosocial functioning in psychosis: systematic review. JMIR Mental Health.

[bb0200] Sharma A. (2024).

[bb0205] Thibaudeau É. (2018). Reliability of two social cognition tests: the combined stories test and the social knowledge test. Psychiatry Res..

[bb0210] Thibaudeau E. (2024). Navigating social cognitive impairments in schizophrenia spectrum disorders: protocol for a pilot pre-post quasi-experimental study for remote avatar-assisted cognitive remediation therapy. JMIR Res. Protoc..

[bb0215] Tracey T.J., Kokotovic A.M. (1989). Factor structure of the working alliance inventory. Psychological Assessment: A journal of consulting and clinical psychology.

[bb0220] Turner-Stokes L. (2009). Goal attainment scaling (GAS) in rehabilitation: a practical guide. Clin. Rehabil..

[bb0225] van der Gaag M. (2013). Development of the Davos assessment of cognitive biases scale (DACOBS). Schizophr. Res..

[bb0230] Vaskinn A., Horan W.P. (2020). Social cognition and schizophrenia: unresolved issues and new challenges in a maturing field of research. Schizophr. Bull..

[bb0235] Vázquez-Campo M. (2016). E-motional training®: pilot study on a novel online training program on social cognition for patients with schizophrenia. Schizophr. Res. Cogn..

[bb0240] Vita A. (2021). Effectiveness, core elements, and moderators of response of cognitive remediation for schizophrenia: a systematic review and meta-analysis of randomized clinical trials. JAMA Psychiatry.

[bb0245] Weinreb S., Li F., Kurtz M.M. (2022). A meta-analysis of social cognitive deficits in schizophrenia: does world region matter?. Schizophr. Res..

[bb0250] Wojtalik J.A. (2023). Predictors of treatment discontinuation during an 18-month multi-site randomized trial of cognitive enhancement therapy for early course schizophrenia. Psychiatry Res..

[bb0255] Wykes T., Crowther A., Reeder C., Medalia A., Bowie C.R. (2016). Cognitive remediation to improve functional outcomes.

[bb0260] Wykes T., Bowie C.R., Cella M. (2024). Thinking about the future of cognitive remediation therapy revisited: what is left to solve before patients have access?. Schizophr. Bull..

[bb0265] Yamada Y. (2022). Pharmacological interventions for social cognitive impairments in schizophrenia: a protocol for a systematic review and network meta-analysis. Front. Psychol..

[bb0270] Yeo H. (2022). A meta-analysis of the effects of social-cognitive training in schizophrenia: the role of treatment characteristics and study quality. Br. J. Clin. Psychol..

